# Discrimination between some *Mycoplasma* spp. and *Acholeplasma laidlawii* in bovine milk using high resolution melting curve analysis

**DOI:** 10.1186/s13104-018-3223-y

**Published:** 2018-02-07

**Authors:** Abd Al-Bar Al-Farha, Kiro Petrovski, Razi Jozani, Andrew Hoare, Farhid Hemmatzadeh

**Affiliations:** 1 Present Address: School of Animal and Veterinary Sciences, Roseworthy Campus, The University of Adelaide, Roseworthy, South Australia 5371 Australia; 2 Mosul Technical Institute, Technical Foundation, Northern Technical University, Mosul, 41000 Iraq; 3 Australian Centre for Antimicrobial Resistance Ecology, The University of Adelaide, Adelaide, South Australia 5000 Australia; 4 Davies Centre, School of Animal and Veterinary Sciences, The University of Adelaide, Roseworthy, South Australia 5371 Australia; 5 Department of Veterinary Clinical Sciences, Tabriz University, Tabriz, 51666-14766 Iran; 6 South East Vets, Mt Gambier, South Australia 5290 Australia

**Keywords:** *Mycoplasma*, Mastitis, Cattle, Milk, *Acholeplasma*

## Abstract

**Objectives:**

This study aimed to provide a rapid, accurate and cost-effective diagnostic real time polymerase chain reaction-high resolution melting curve assay (PCR-HRM) to identify and distinguish between four different mycoplasmas and *Acholeplasma laidlawii* isolated at cow-level from a single commercial dairy farm in South Australia. One set of genus-level universal primers was designed targeting the 16S ribosomal RNA gene.

**Results:**

Real time PCR-HRM analysis was able to identify and distinguish between five different mollicutes, namely *A. laidlawii*, *M. arginini*, *M. bovirhinis*, *M. bovis* and uncultured *Mycoplasma*. Results were confirmed through sequencing. Our developed assay provides rapid and accurate screening for *Mycoplasma* mastitis detection.

## Introduction

*Mycoplasma* mastitis is of emerging significance worldwide, posing significant economic impacts on the dairy industry. Early detection of *Mycoplasma* mastitis is important to disease control strategies [1]. Several *Mycoplasma* spp. are mainly responsible for mastitis, including *M. bovis*, *M. bovoculi*, *M. alkalescence*, *M. alvi*, *M. bovigenetalium*, *M. bovirhinis*, *Mycoplasma species bovine group 7, M. californicum, M. dispar, M. canis, M. verecundum, M. canadense* and *M. mycoides* subsp. *mycoides* [2]. *Acholeplasma* spp. may be isolated from milk either as a contaminant [3] or as a co-invader with other mycoplasmas [4, 5]. Conventional microbial culture of mollicutes can be laborious and time-consuming with a variety of species-specific growth requirements [6]. Misdiagnosis of *Mycoplasma* using serological detection is common due to the lag period required for antibody formation. Therefore, a rapid and accurate diagnostic assay is required for screening of mycoplasma in dairy herds. High resolution melting curve analysis (HRM) has been recently developed and widely used for phenotyping at strain or species-level of various organisms including mycoplasmas [7, 8]. However, field isolates of mastitis related mycoplasmas and other milk environmental mollicutes have not been assessed previously using this method.

The aim of this study was to provide a suitable diagnostic real time polymerase chain reaction-high resolution melting curve analysis (PCR-HRM) to identify and distinguish between five different mollicutes isolated at cow-level from a single commercial dairy farms in South Australia.

## Main text

### Methods

Samples were selected based on conventional PCR findings of a previous study conducted on single commercial dairy farm in South Australia. This farm had a history of repeated mastitis treatment failure with high somatic cell count (SCC) and poor response to antimicrobials [4]. Six isolates for each of the following species were selected in this study: *A. laidlawii*, *M. arginini*, *M. bovirhinis*, *M. bovis* and uncultured mollicutes. One set of genus-level universal primers targeting the 16S rRNA gene was designed for real time-PCR. Forward primer Mol-F: GGCGAAYGGGTGAGTAACAC and reverse primer Mol-R: CATHGYCTTGGTRRGCYNTTA. The real time PCR mixture was prepared using HRM kit AccuMelt HRM SuperMix (Quantabio, Australia). DNA amplification was conducted in a 96 microplate (Illumina, San Diego, CA, USA). Each well contained 10 µL reaction solution of 5 µL HRM SuperMix, 1 µL DNA template (approximately 20 ng), 1 µL each primer (0.2 nmol) and 2 µL nuclease free water (Qiagen, Germany). The reaction was conducted using an Illumina Thermal Cycler with pre-heating activation for 2 min followed by 40 PCR cycles of three steps: denaturation at 95 °C for 15 s, annealing at 60 °C for 45 s, then extension at 72 °C for 15 s. HRM was performed at 55–95 °C at the rate of 0.1 °C. Results were analysed via EcoStudy software (version 5.0, Illumina). PCR products were subject to electrophoresis in 1.5% agarose gels and visualised by staining with Gel Red. PCR products from the 16S rRNA gene were submitted to the Australian Genome Research Facility Ltd (AGRF, Adelaide, South Australia) for Sanger sequencing. Each fragment was sequenced in forward and reverse directions. To reconstitute the sequence, forward and reverse sequences were edited and assembled using BioEdit package v.7.0.4.1. Edited sequences were blasted against existing sequences in GeneBank using the basic local alignment search tool (BLAST) (http://blast.ncbi.nlm.nih.gov/Blast.cgi) and nucleotide sequences from relevant *Mycoplasma* strains were used as reference strains for nucleotide alignments using ClustalW program version 2.

### Results

**Fig. 1:**
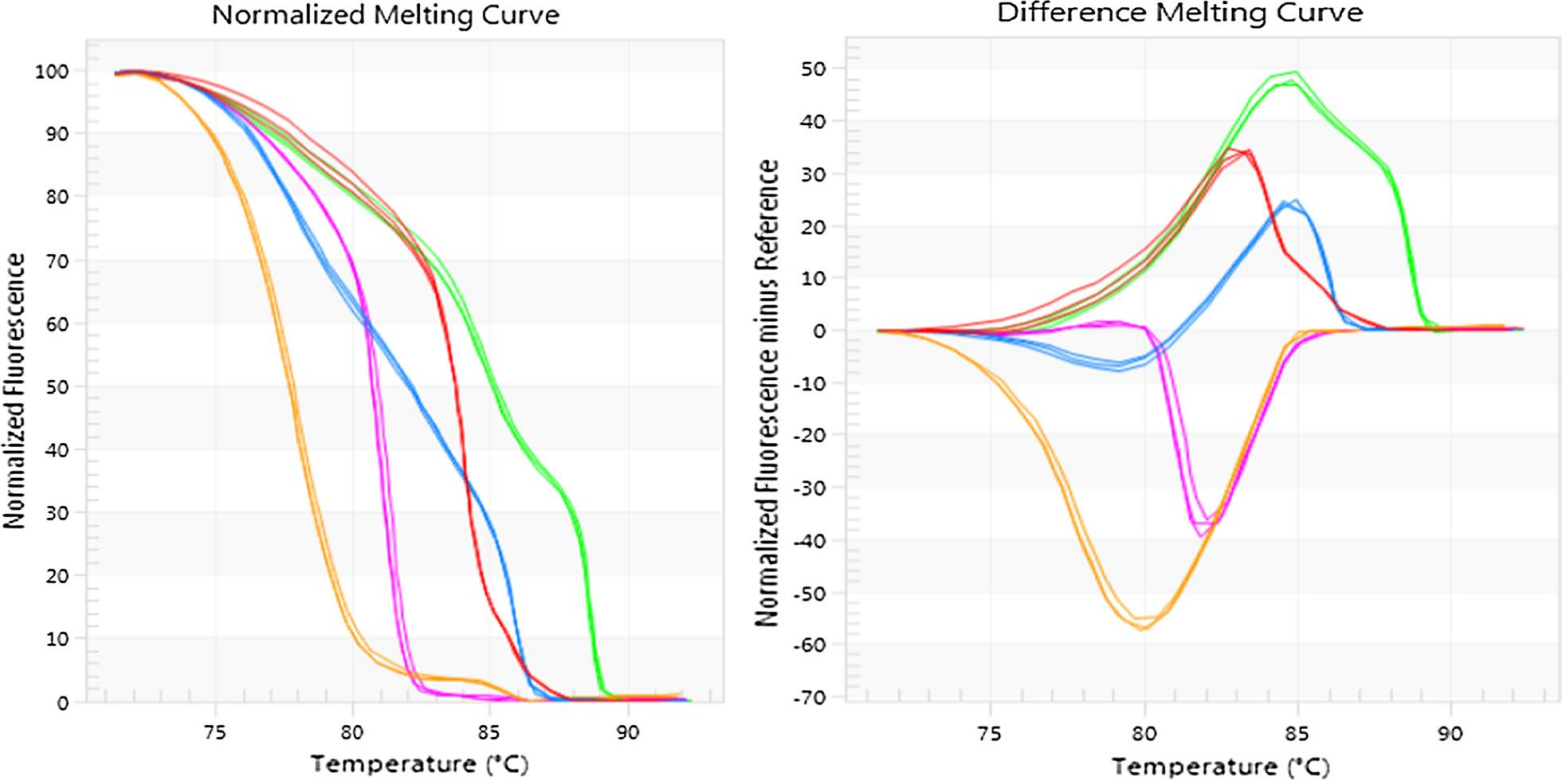
Normalised and different high resolution melting curves generated by EcoStudy software of five different mollicutes isolated from bovine milk: purple—*Acholeplasma laidlawii*, green—*Mycoplasma arginini*, blue—*M. bovirhinis*, yellow—*M. bovis*, red—uncultured *Mycoplasma* spp.

Five different mollicutes, *A. laidlawii, M. arginini, M. bovirhinis, M. bovis* and uncultured mollicutes, produced normalised and derivative melt curves (Fig. 1). *A. laidlawii* (Accession No. LC201977.1) generated one melting peak at 81.2 °C, *M. arginini* (Accession No. LC158832.1) generated two melting peaks at 88.5 and 84.7 °C. *M. bovirhinis* (Accession No. AP018135.1) generated three melting peaks at 85.7, 77.6 and 88.2 °C. *M. bovis* (Accession No. KX462439.1) generated two melting peaks at 77.6 and 85.2 °C. Uncultured *Mycoplasma* spp. (Accession No. LT679634.1) generated one melting peak at 83.9 °C.

### Discussion

While conventional culture, the traditional method for mollicute detection, imposes technical challenges in distinguishing between milk pathogenic and saprophytic mollicutes, our study indicated that real time PCR-HRM assay provides a sensitive, rapid and cost-effective screening method to identify and discriminate between some pathogenic and environmental mollicutes isolated from milk DNA. Effects of some of these mollicutes on SCC and milk composition have been previously studied [4]. We considered SCC as the crucial factor that discriminates between contagious and environmental mollicutes. *M. bovis* was widely reported as a main mastitis causing *Mycoplasma* [9, 10]. Inconsistent results have been reported regarding involvement of *M. arginini, M. bovirhinis* and *A. laidlawii* in bovine mastitis, particularly with co-infection *Mycoplasma* mastitis [5, 11, 12]. However, several studies indicate these mollicutes are not significant pathogens [3, 13].

Melting profile, introduced in 2002, is widely used for genotyping a wide range of microorganisms [14–17]. HRM-based assay describes correlation between temperature and DNA extent of denaturation [18]. The variety of melting temperatures for different species is attributed to DNA length, sequencing and GC content [14]. In summary, as an alternative to sequencing, our developed real time PCR-HRM assay offered a rapid, low-cost and simple discriminative method to distinguish between some mastitis causing pathogenic mycoplasmas and other saprophytic mollicutes in bovine milk. This method was useful for screening of *Mycoplasma* mastitis and can be extended to identify more mollicutes species.

### Limitations

One of the limitations of HRM-based analysis in *Mycoplasma* mastitis detection is the inability to detect co-infection cases due to amplimer concentration differences and the requirement of separation each individual amplicon Tm [18]. Primers used in this study were designed to target more spp. of major *Mycoplasma*-causing mastitis in dairy herds. However, in this study, we used only field isolates of five different mollicutes.

## Abbreviations

HRM: high resolution melt
PCR: polymerase chain reaction
SCC: somatic cell count
